# Integrative analysis for the discovery of lung cancer serological markers and validation by MRM-MS

**DOI:** 10.1371/journal.pone.0183896

**Published:** 2017-08-24

**Authors:** Jihye Shin, Sang-Yun Song, Hee-Sung Ahn, Byung Chull An, Yoo-Duk Choi, Eun Gyeong Yang, Kook-Joo Na, Seung-Taek Lee, Jae-Il Park, Seon-Young Kim, Cheolju Lee, Seung-won Lee

**Affiliations:** 1 Center for Theragnosis, Korea Institute of Science and Technology, Seongbuk-gu, Seoul, Korea; 2 Department of Biochemistry, College of Life Science and Biotechnology, Yonsei University, Seodaemun-gu, Seoul, Korea; 3 Department of Thoracic and Cardiovascular Surgery, Chonnam National University Hwasun Hospital, Hwasun-gun, Jeollanam-do, Korea; 4 KIST School, Korea University of Science and Technology, Daejeon, Korea; 5 Department of Anatomy, Chonnam National University Medical School, Hwasun-gun, Jeollanam-do, Korea; 6 Department of Pathology, Chonnam National University Hospital, Dong-gu, Gwangju, Korea; 7 Animal Facility of Aging Science, Korea Basic Science Institute, Buk-gu, Gwangju, Korea; 8 Personalized Genomic Research Center, Korea Research Institute of Bioscience and Biotechnology, Daejeon, Korea; 9 Department of Functional Genomics, University of Science and Technology, Daejeon, Korea; Peking University People's Hospital, CHINA

## Abstract

Non-small-cell lung cancer (NSCLC) constitutes approximately 80% of all diagnosed lung cancers, and diagnostic markers detectable in the plasma/serum of NSCLC patients are greatly needed. In this study, we established a pipeline for the discovery of markers using 9 transcriptome datasets from publicly available databases and profiling of six lung cancer cell secretomes. Thirty-one out of 312 proteins that overlapped between two-fold differentially expressed genes and identified cell secretome proteins were detected in the pooled plasma of lung cancer patients. To quantify the candidates in the serum of NSCLC patients, multiple-reaction-monitoring mass spectrometry (MRM-MS) was performed for five candidate biomarkers. Finally, two potential biomarkers (BCHE and GPx3; AUC = 0.713 and 0.673, respectively) and one two-marker panel generated by logistic regression (BCHE/GPx3; AUC = 0.773) were identified. A validation test was performed by ELISA to evaluate the reproducibility of GPx3 and BCHE expression in an independent set of samples (BCHE and GPx3; AUC = 0.630 and 0.759, respectively, BCHE/GPx3 panel; AUC = 0.788). Collectively, these results demonstrate the feasibility of using our pipeline for marker discovery and our MRM-MS platform for verifying potential biomarkers of human diseases.

## Introduction

Owing to its high metastatic potential and high mortality rate, lung cancer is one of the leading causes of cancer-related deaths worldwide and is broadly divided into two classes, small cell lung cancer (SCLC) and non-small cell lung cancer (NSCLC). NSCLC, which constitutes approximately 80% of all diagnosed lung cancers, consists of two major subtypes: adenocarcinoma (ADC) and squamous cell carcinoma (SQC). Every year, more than 900,000 deaths from ADC and SQC combined are reported worldwide [1]. Detection of NSCLC via markers is critical in improving prognoses and survival rates. Although serological biomarkers can be analyzed relatively easily and economically, the discovery of biomarkers using plasma and serum samples is challenging due to the high complexity and wide dynamic range of the plasma proteome. Enormous efforts have been made to discover biomarkers using plasma samples from NSCLC patients [2], but unfortunately, clinical use of those markers might be limited.

High-throughput omics-based technologies have been widely used in the past decade, and many groups have researched the characterization and dysregulation of tumor genomes and differentially expressed proteins and their mechanisms. The resulting genomic, transcriptomic, and proteomic data have been deposited in public databases [3]. Technological developments in areas such as proteomics, bioinformatics, and biostatistics, coupled with knowledge from the map of the human genome and the human proteome, are expected to drive further improvements within healthcare, specifically with biomarkers for diagnoses and prognoses. Among lung cancer studies, many have searched for potential NSCLC markers using various biological specimens, including cell lines, tissues, and serum/plasma at the discovery stage [2, 4–9]. The resulting candidates have been evaluated in human serum/plasma using antibody-based assays such as ELISA for their diagnostic potential as noninvasive biomarkers. However, ELISA kits are expensive and time-consuming and allow measurement of only one protein at a time. Also, the availability of high-quality commercial ELISA kits is dependent on antibody quality [10]. Recent advances in MS-based quantification, especially selected reaction monitoring (SRM) or multiple reaction monitoring (MRM), enable accurate measurements of target proteins without antibodies [11–13]. High selectivity and specificity, attained through the use of two mass-filtering quadrupoles, have facilitated selection of target transitions from complex samples such as plasma/serum and allowed reproducible analyses.

In this study, we hypothesized that if some proteins show quantitative changes in cancer tissues compared to normal tissues and are present in the cell secretome, then there would be a better chance of detecting those proteins in plasma/serum. We challenged this hypothesis with NSCLC integrative tissue transcriptomes and cell secretomes, and the resulting candidates were verified using MRM-MS and ELISA to measure the concentrations of candidate proteins in plasma. To find differentially expressed genes (DEGs) in NSCLC tissues, we analyzed transcriptome data from 9 datasets deposited in the Gene Expression Omnibus (GEO). In addition, secretomes, which have limited complexity compared to serum and plasma, were profiled from six lung cancer cell lines. Overlapping potential candidates from DEGs and secreted proteins were confirmed in pooled plasma by LC-MS/MS. MRM-based target quantification was applied to evaluate the candidates using clinically collected sera including 23 healthy controls and 23 NSCLC cases (16 ADCs and 7 SQCs). Finally, two potential biomarkers (BCHE and GPx3; AUC = 0.713 and 0.673, respectively) and one two-marker panel (BCHE/GPx3; AUC = 0.773) were verified by MRM-MS. An additional validation study was performed on the two biomarkers (GPx3 and BCHE) using ELISA-based protein quantitation in an independent set of samples (50 healthy controls and 50 NSCLC patients). The GPx3 and BCHE values were significantly lower in the NSCLC group compared with the healthy controls (BCHE and GPx3; AUC = 0.630 and 0.759, respectively, BCHE/GPx3 panel; AUC = 0.788).

## Materials and methods

### Mining microarray data from the gene expression omnibus

Human arrays can be obtained from GEO at the National Center for Biotechnology Information (NCBI), which has emerged as the leading fully public repository for gene expression data (http://www.ncbi.nlm.nih.gov/geo/). Nine datasets (GEO accession numbers GSE12667 [14], GSE10245 [15], GSE18842 [16], GSE10445 [17], GSE19188 [18], GSE10799 [19], GSE19804 [20], GSE27262 [21], and GSE31210 [22]) were selected to compare mRNA expression between lung cancer and normal tissues as raw CEL files were available for those nine data sets produced by using the same platform (GPL570; Affymetrix Human Genome U133 Plus 2.0 Array). The results are summarized in Table 1. First, raw CEL files were downloaded from the GEO site, and rma normalization was performed using the total of 887 cel files using affy package [23, 24]. Then, heteroscedastic t-tests were performed to determine the statistical significance of the data (p < 0.0001) and false discovery rates (FDRs) were calculated to correct the P-value (q < 0.001) [25].

**Table 1:** Summary of 9 transcriptome datasets obtained from the GEO.

Accession number	Title	# of samples (tumor/normal)	reference
GSE10245	Non-small lung cancer subtypes: adenocarcinoma and squamous cell carcinoma	58 (58/0)	[14]
GSE10445	Merlion lung cancer study	72 (72/0)	[15]
GSE10799	Gene expression profile of lung tumors	19 (16/3)	[16]
GSE12667	Discovery of somatic mutations in lung adenocarcinomas	75 (75/0)	[17]
GSE18842	Gene expression analysis of human lung cancer and control samples	91 (46/45)	[18]
GSE19188	Expression data for early stage NSCLC	156 (91/65)	[19]
GSE19804	Genome-wide screening of transcriptional modulation in non-smoking female lung cancer in Taiwan	120 (60/60)	[20]
GSE27262	Gene expression profiling of Non-small cell lung cancer in Taiwan	50 (25/25)	[21]
GSE31210	Gene expression data for pathological stage I-II lung adenocarcinomas	246 (226/20)	[22]

### Preparation of secretome

The lung cancer cell lines A549, Calu-1, H1299, H23, H460 and H520 were used and cultured in RPMI 1640 (Gibco, Rockville, MD, USA) supplemented with 10% FBS (Gibco) and 1% penicillin/streptomycin (Gibco) at 37°C in a humidified 95% air, 5% CO_2_ incubator. Cells were grown to approximately 70% confluence and rinsed carefully three times with serum-free medium (SFM) at room temperature. Then, the cells were incubated in SFM at 37°C for 12 h to minimize the release of cytosolic proteins into the surrounding medium due to cell death. After incubation, the conditioned media were carefully collected and 2 mM PMSF and 1 mM EDTA were added as protease inhibitors. Floating cells and cellular debris were removed by centrifugation (400 × g, 10 min, 4°C), followed by sterile filtration (pore size: 0.22 μm, Millipore, MA, USA). The media were concentrated and exchanged into buffer consisting of 8 M urea, 75 mM NaCl, and 50 mM Tris (pH 8.2) by ultrafiltration using Amicon Ultra-15 centrifugal filter devices (Millipore).

### Plasma and serum

For all blood preparations, 3 mL of blood was collected in an EDTA tube and the plasma was prepared as suggested by the HUPO Plasma Proteome Project [26]. The plasma samples for prescreening were collected at Yonsei Severance Hospital (Seoul, Korea) and authorization to use the samples for research purposes was obtained from the institutional review board (IRB). Plasma samples pooled from 10 lung cancer patients and 10 healthy controls were used. The top 12 abundant proteins (α1-acid glycoprotein, fibrinogen, α1-antitrypsin, haptoglobin, α2-macroglubulin, IgA, albumin, IgG, apolipoprotein A-I, IgM, apolipoprotein A-II and, transferrin) in the plasma were depleted using Pierce™ Top 12 Abundant Protein Depletion Spin Columns (Pierce, Rockford, IL, USA).

The serum specimens used for the MRM study were obtained from 23 patients with NSCLC (16 adenocarcinomas and 7 squamous cell carcinomas sampled before initiation of treatment at Chonnam National University Hwasun Hospital) and from 23 normal healthy controls. For an ELISA-based validation, an independent set of NSCLC cases (n = 50; all new) and controls (n = 50; 41 samples were new and the other 9 samples were from the MRM controls) was selected. Clinical information was obtained by chart review and all diagnoses were confirmed histologically by surgical biopsy. The Institutional Board of Review of Chonnam National University Hwasun Hospital approved this study and the need for consent was waived by the ethics committee. Serum samples were immediately placed on ice for transport to the laboratory where they were centrifuged, aliquoted, and immediately frozen at -80°C until use.

### Protein digestion by trypsin

For in-solution digestions, all samples (~100 μg), such as the secretome from cell lines, plasma, and serum, were reduced with 5 mM DTT (Sigma, St. Louis, MO, USA) at 30°C for 1 h and alkylated with 14 mM iodoacetamide (Sigma, St. Louis, MO, USA) at 30°C for 1 h. The sample was diluted five times to decrease the urea concentration to 1.6 M in solution and CaCl_2_ (Sigma) was added to a final concentration of 1 mM. The protein mixture was digested by sequencing grade modified trypsin (Promega, Madison, WI, USA) at 37°C for 16 h (enzyme:protein = 1:50). Tryptic peptides were desalted with a C_18_ spin column (Thermo Scientific, Waltham, MA, USA) and further fractionated.

For in-gel digestions, pooled plasma was reduced in the gels with 20 mM DTT at 56°C for 1 h, followed by alkylation with 50 mM iodoacetamide in 25 mM ammonium bicarbonate for 1 h in the dark. After treatment, the reagents were removed and gel pieces were washed with 50 mM ammonium bicarbonate and then dehydrated in acetonitrile. The dried gel pieces were then rehydrated in a solution of trypsin (Promega) in 25 mM ammonium bicarbonate for 16 h. Tryptic peptides were extracted from the gel with 50% acetonitrile in 5% formic acid followed by 70% acetonitrile in 5% formic acid. All digests were desalted with a C_18_ spin column, dried *in vacuo*, and stored at -80°C until use.

#### Fractionation after in-solution digestion

Tryptic digests from the secretome were further separated into 12 fractions based on peptide isoelectric points using an OFFGEL 3100 fractionator (Agilent Technology, Santa Clara, CA, USA). All fractions were desalted with C_18_ spin columns (Thermo Scientific), dried *in vacuo*, and stored at -80 dried ntific), dried based on peptide isoelectric points using an OFFGEL 3100 fractionator (Agilent Technology, Santa Clara, CA, USA). All fractions were desalted with CAl The fractionations were performed with XBridge C-18 columns (i.d. 4.6 mm; length 250 mm; pore size 130 130 pore size 130 ze 130 ectric points using an OFFGEL 3100 fractionator (Agilent Technology, Santa Clara, CA, UmM ammonium formate (pH 10) and mobile phase B was 10 mM ammonium formate in 90% acetonitrile (pH 10). Plasma digests were dissolved in 125 μL of mobile phase A and then manually injected into a 100-μL sample loop. A gradient of 5anually injected into a 100-d in 125 in 125 ed in 125 5 OFFGEL 3 for 10 min, and 45–90% phase B for 15 min was applied, followed by washing the column with 100% and 50% mobile phase B for 30 min each. Throughout the fractionation process, eluates were collected every minute on a 96-well plate in the Frac-950 fraction collector of an AKTA Explorer (GE Healthcare Life Science) [12]. In total, 96 fractions, 8 rows (Ally × 12 columns (1fractions, 8 rows (Ally injected into a 100-d in 125 in 125 ed in 125 5 OFFGEL 3 for 10 min, and lso traced the surrogate peptide during high-pH RPLC by monitoring the UV absorbance of the eluate at 215 nm. The fractions selected for the next step of LC–SRM/MS were dried and stored at –20°C until use.

### Liquid chromatography and tandem mass spectrometry (LC-MS/MS)

The peptide samples were reconstituted in 0.4% acetic acid. An aliquot (~1 μg) was then injected into a reversed-phase Magic C_18_aq column (15 cm × 75 μm, 200Å, 5U). The column was pre-equilibrated with 95% buffer A (0.1% formic acid in water) and 5% buffer B (0.1% formic acid in acetonitrile) on an Agilent 1200 HPLC system (Agilent Technology). The peptides obtained from the secretome were eluted at a flow rate of 0.4 μL/min across the analytical column with a linear gradient of 5–40% buffer B over 90 min for each fractionated peptide. The HPLC system was coupled to an LTQ-XL mass spectrometer (Thermo Scientific, San Jose, CA). The ESI voltage was set to 1.9 kV, the capillary voltage to 30 V, and the temperature of the heated capillary to 250°C. The MS survey was scanned from 300 to 2,000 m/z, followed by three data-dependent MS/MS scans with the following options: isolation width, 1.5 m/z; normalized collision energy, 25%; dynamic exclusion duration, 180 s. All experiments were conducted in duplicate runs and all data were acquired using Xcalibur software v2.0.7.

Analytes obtained from plasma were analyzed using an LTQ XL-Orbitrap mass spectrometer (Thermo Scientific). The spray voltage was set to 1.9 kV and the temperature of the heated capillary was set to 250°C. Survey full-scan MS spectra (300–2000 m/z) were acquired from the Orbitrap with one microscan and a resolution of 100,000, allowing preview mode for precursor selection and charge-state determination. MS/MS spectra of the 10 most intense ions from the preview survey scan were acquired concurrently from the ion-trap for full-scan acquisition in the Orbitrap with the following options: isolation width, 10 ppm; normalized collision energy, 35%; dynamic exclusion duration, 30 s. Precursors with unmatched charge states were discarded during data-dependent acquisition. Singly charged precursors were also excluded. Data were acquired using Xcalibur software v2.0.7.

### Analysis of mass spectrometric data

The acquired MS/MS spectra from the cell secretome were searched using SEQUEST (TurboSequest version 27, revision 12) against the composite UniProtKB database (released in March 2012), including the human experimentally validated FBS UniProtKB database [27]. Two trypsin-missed cleavages were allowed and the peptide mass tolerances for MS/MS and MS were set to ± 0.5 and ± 2 Da, respectively. Other options used for SEQUEST were fixed modification of carbamidomethylation at cysteine (+ 57.02 Da) and variable modification of oxidation at methionine (+ 15.99 Da). Peptide assignment and validation were performed using the Trans-Proteomic Pipeline (TPP, version 4.0, http://www.proteomecenter.org). The SEQUEST search output was used as an input for TPP analyses. The MS/MS spectra acquired from plasma samples were analyzed using SEQUEST in Proteome Discoverer 1.4 (Thermo Fisher Scientific, version 1.4.0.288). The search parameters were identical to the abovementioned parameters. For the validation of peptide sequence matching (PSM), Percolator was used to calculate q-values as measures of statistical confidence for each PSM. We filtered the output with q < 0.01.

### Bioinformatics for data mining

The identified secretory proteins were analyzed using ProteinCenter (Proxeon Bioinformatics, http://www.cbs.dtu.dk/services). We submitted several proteins in one FASTA format file for each program. We used SignalP (version 4.0, http://www.cbs.dtu.dk/services/SignalP4.0) to predict the presence of signal peptides in the identified proteins [28]. The SecretomeP program (version 2.0, http://www.cbs.dtu.dk/services/SecretomeP2.0) was used to predict the possibility of nonclassical protein secretion [29]. In addition, the TMHMM program (version 2.0, http://www.cbs.dtu.dk/services/TMHMM2.0) was used to predict transmembrane helices in integral membrane proteins [30]. All of the secreted proteins were further analyzed using the Plasma Proteome Database (PPD, http://www.plasmaproteomedatabase.org). Ingenuity Pathway Analysis (IPA, Ingenuity system, http://www.ingenuity.com) was used to determine the subcellular localizations and biological functions of the proteins.

### Stable isotope standard (SIS) synthetic peptides

The MRM assay panel consisted of 8 light peptides as surrogates for 5 plasma proteins that were obtained from prescreening LC-MRM/MS data with the corresponding 8 SIS peptides as internal standards. Selected peptides were noted for doubly or triply charged precursor ions to ensure stable and reproducible detection for quantification. Synthetic SIS peptides incorporating C-terminal [^13^C_6_, ^15^N_2_] lysine and [^13^C_6_, ^15^N_4_] arginine were obtained from 21st Century Biochemical (Marlboro, MA, USA). For each peptide, amino acid analysis (AAA) was performed by the vendor. These peptides were resolubilized in 20% acetonitrile with 0.1% formic acid at a similar scale as that expected for target proteins in plasma. After pooling the 11 SIS peptides, the mixture was spiked into plasma prior to tryptic digestion.

### Multiple reaction monitoring mass spectrometry (MRM-MS)

The LC-MRM-MS assay was executed by two-column switching on a nanoLC-MRM-MS system. The LC system consisted of an Eksigent nanoLC-Ultra 2D Plus interfaced with a NanoFlex system. Mobile phase A and mobile phase B consisted of water (solvent A) and acetonitrile (solvent B), respectively, both containing 0.1% formic acid. Plasma sample digests were reconstituted with 22.5 μL of 2% acetonitrile and 0.1% formic acid. Each sample was picked up with a 1-μL sample loop and separated using a Nano cHiPLC ReproSil-Pur C18-column (i.d. 75 μm, length 15 cm, pore size 120 Å, particle size 3 μm; Eksigent Technologies, Dublin, CA) with a flow rate of 300 nL/min. The columns were maintained at 40°C. Due to the use of a two-column switching method, the sample was loaded onto the column being regenerated (i.e., the column where flow was not directed to the mass spectrometer) as follows: an M-shaped gradient of mobile phase B (5–90–5–90–5%) for 10 min followed by re-equilibration with 5% B for 40 min. The sample was injected onto the loading column at 30 min. The column was switched (i.e., flow was directed to the mass spectrometer) and the sample was separated by the following elution method: gradient from 5% to 10% B over 4 min, gradient from 10% to 25% B over 30 min, gradient from 25% to 60% B over 3 min, hold 60% B for 3 min, gradient from 60% to 5% B over 1 min, and hold 5% B for 9 min. The LC system was coupled to a 5500 Qtrap mass spectrometer by a nanoelectrospray ion source (SCIEX, Foster City, CA). MS detection was carried out in positive MRM mode with the following parameters: ion spray voltage of 2200 V, curtain gas at 10 psi, nebulizer gas at 30 psi, resolution at 0.7 Da (unit resolution) for Q1/Q3, interface temperature at 150°C, and scan mass range of m/z > 300–1250. The collisional energy (CE) and declustering potential (DP) were optimized by direct infusion using Turbospray (Sciex, Foster City, CA). Quantification was performed using the nonscheduled MRM mode with a dwell time of 20 ms and a cycle time of 2.4 s [30]. The raw data were deposited in the PeptideAtlas database (accession ID, PASS00767).

### Analysis of MRM-MS data

Analyst software (Version 1.5.2, ABSciex) was used to generate MRM-MS data and optimized MRM parameter data (*.wiff). Skyline (version 2.6.0) was used to analyze MRM results from extracted ion chromatograms (XICs) [31]. Endogenous peptides were identified and quantified with the corresponding SIS peptides. We chose the single quantitative transition that had no interference and achieved the most sensitivity. Other transitions were used for peak assignments. Marker candidate proteins were statistically analyzed using Excel 2010 (version 14.0, Microsoft Office) and R software (version 3.1.0).

### ELISA-based quantification of proteins in serum specimens

The serum levels of GPx3 and BCHE were determined using commercial ELISA kits (AdipoGen, Inc., Seoul, Korea; R&D Systems, Inc., MN, USA, respectively). Each microtiter plate was coated with a human GPx3 specific polyclonal antibody or a human BCHE specific monoclonal antibody. For GPx3, each patient serum sample was prepared at dilution 1:250, 100 μL of which was added to the wells and incubated for 1 h at 37°C. After washing the plate three times, 100 μL of the primary detection antibody was added. After incubation at 37°C for 1 h, the plate was washed three more times and 100 μL of the secondary detection antibody was added. This was followed by an additional 1-h incubation at 37°C. After washing the plate five times, 100 μL of the substrate solution was added. The plate was then read within 20 min at a wavelength of 450 nm using a VERSAmax microplate reader (Molecular Devices, Sunnyvale, CA). For BCHE, each serum sample was diluted 1:2000, 50 μL of which was added to the wells. The plates were incubated at room temperature for 2 h on a horizontal orbital microplate shaker set at 500 rpm. After washing the plate three times, 200 μL of the detection antibody was added and incubated for 2 h at room temperature on a shaker. After washing three times, 200 μL of substrate solution was added to each well. This was followed by a 30-min incubation at room temperature in the dark. The absorption at 450 nm was read in an Infinite M200 Pro microplate reader (TECAN, Männedorf, Switzerland). All experimental procedures were performed in duplicate, and measurements were averaged.

### Statistical analysis

Analysis of ELISA data was performed using SOFTMax Pro version 5 software (Molecular Devices, Sunnyvale, CA). All MRM-MS and ELISA values are expressed as the mean ± SD. The Mann-Whitney test was used to compare age, BCHE and GPx3 levels between the control and NSCLC groups. We used a chi-square test to assess differences in sex, history of smoking, hypertension (HTN), and diabetes mellitus (DM) between serum sample groups. The sample size computation for the ELISA validation was done using G*Power 3.1.9.2 [32]. Multivariate logistic regression analysis was used to assess the association of biomarkers with the risk of lung cancer. Spearman correlation analysis was performed to test the association of biomarker levels with the stage of lung cancer. The AUC was estimated by receiver operating characteristics curve (ROC) analysis. A p-value < 0.05 was considered statistically significant. All statistical analyses except for sample size computation were performed with SPSS Statistics 21 (IBM, Armonk, United States).

## Results

### Quantitative analysis of GEO data

**Fig 1:**
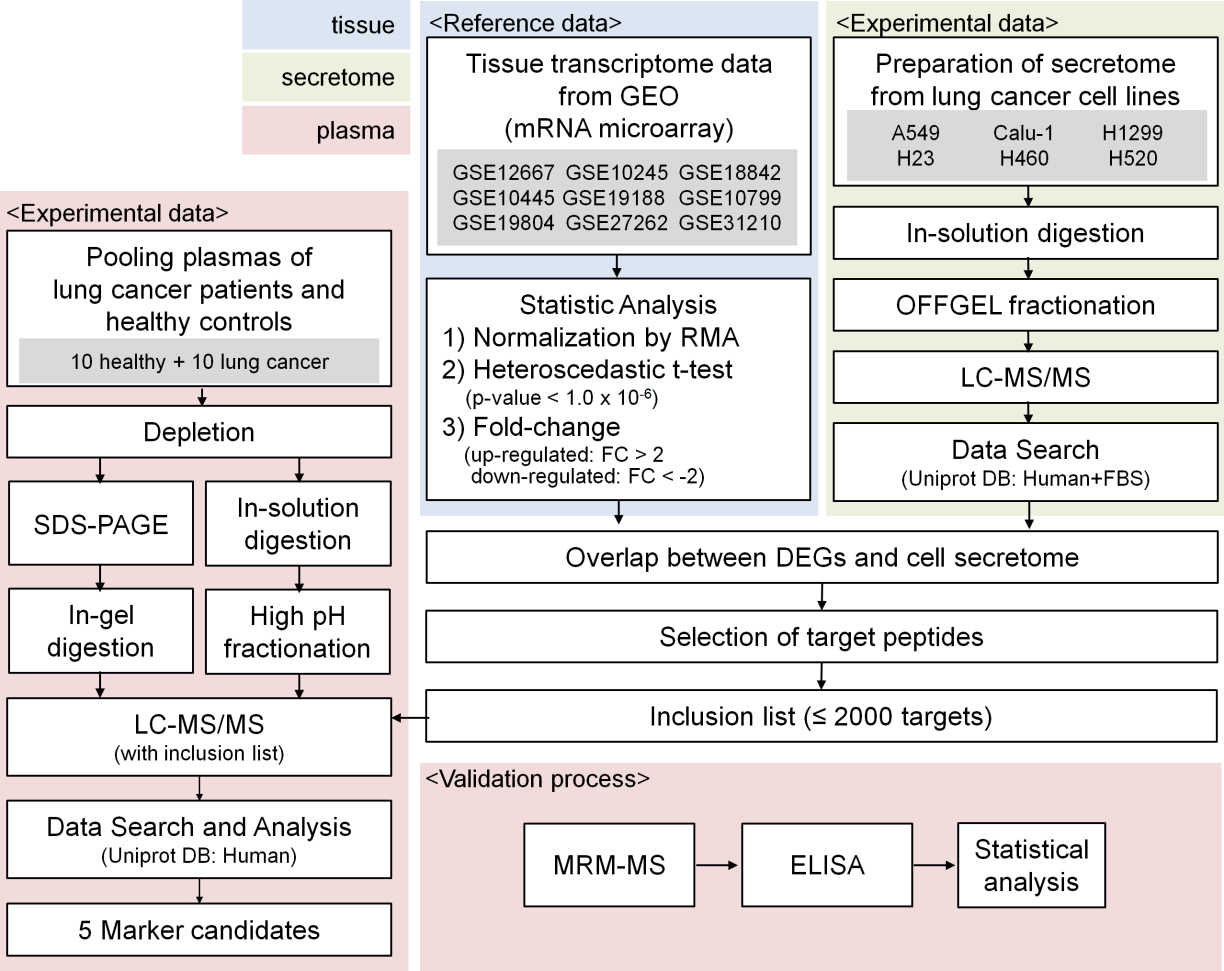
Schematic workflow.

**Fig 2:**
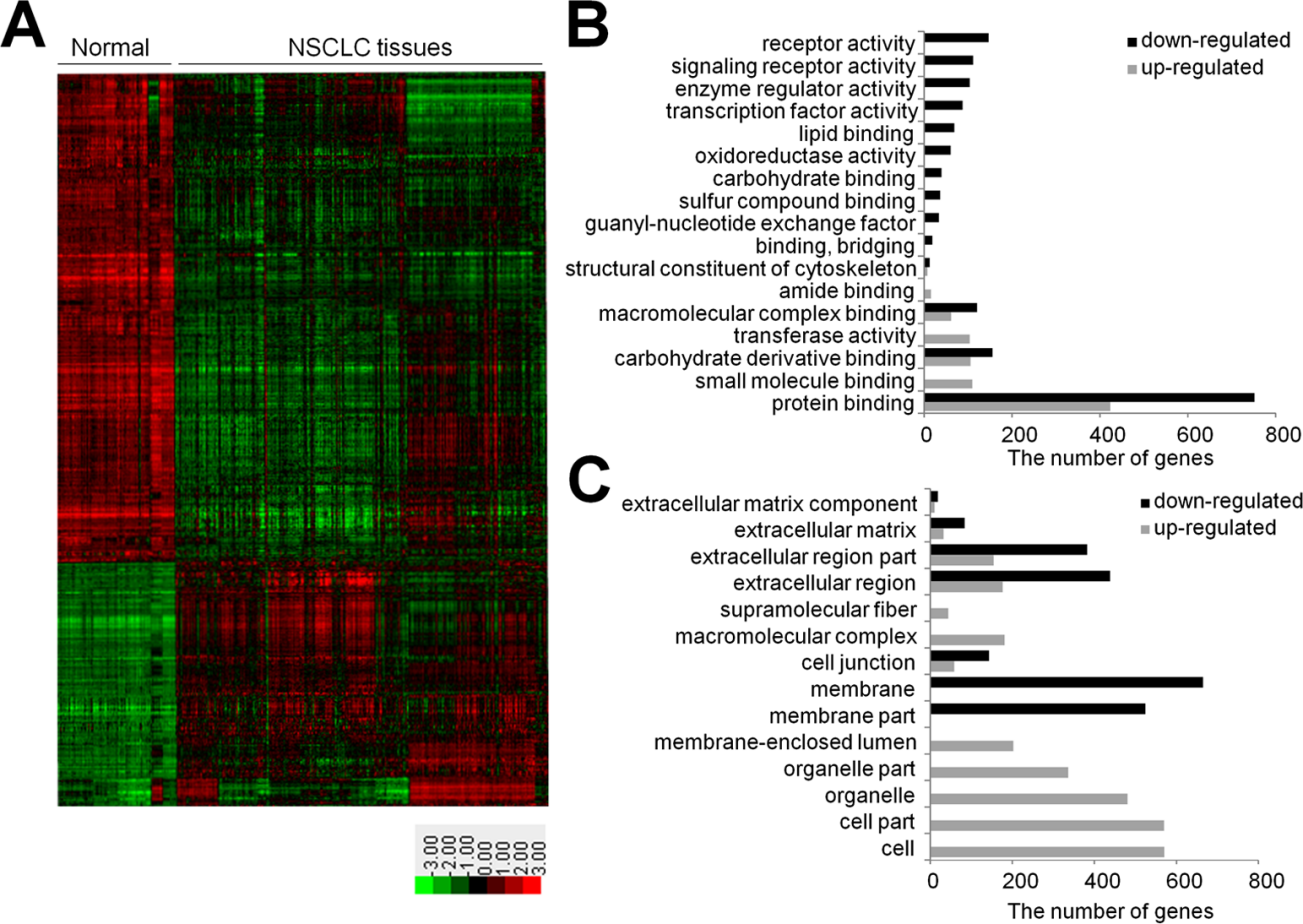
Analyses of 9 transcriptome datasets from the GEO. **(A)** A heat map of 2,696 differentially expressed probes between tumor (n = 669) and non-tumor tissues (n = 218) collected from 9 GEO data sets (p-values < 1 x 10^−6^ and over two-fold changes; red: up-regulation; green: down-regulation) **(B)** Classification of DEGs based on their molecular function as suggested by DAVID. **(C)** Subcellular locations of DEGs (grey: up-regulated genes in NSCLC; black: down-regulated genes in NSCLC).

A schematic for the experimental procedure used in our study is shown in Fig 1. The starting point was the analysis of gene-expression data. Lung cancer transcriptome data were obtained from 9 GEO datasets (GSE12667 [14], GSE10245 [15], GSE18842 [16], GSE10445 [17], GSE19188 [18], GSE10799 [19], GSE19804 [20], GSE27262 [PMID22726390], and GSE31210 [PMID 23028479]) and used for the discovery of NSCLC-specific genes (Table 1). The analysis included 54,613 probes from 218 normal lung tissues and 669 lung cancer tissues (S1 Table). By applying global normalization to all datasets, heteroscedastic t-test p-values < 1 x 10^−6^ and over two-fold changes, we gathered a total of 1,890 DEGs from 2,696 probes, of which 683 genes were up-regulated (maximal log fold-change; 4.33) and 1,207 genes were down-regulated (minimal log fold-change; -5.03) in lung cancer tissues relative to non-tumor tissues. A hierarchical cluster analysis of the DEGs is presented as a heat map in Fig 2A. The up-regulated genes in cancer tissues were involved in protein binding, small molecule binding, carbohydrate derivative binding and transferase activity, while the down-regulated genes were concerned with protein binding, carbohydrate derivative binding, receptor activity, macromolecular complex binding and signaling receptor activity (Fig 2B). The up-regulated genes were primarily compartmentalized in the cell and organelle parts and some were in the extracellular region, while the down-regulated genes were in the extracellular region and membrane part (Fig 2C)

### Identification of lung cancer cell secretomes by LC-MS/MS

**Fig 3:**
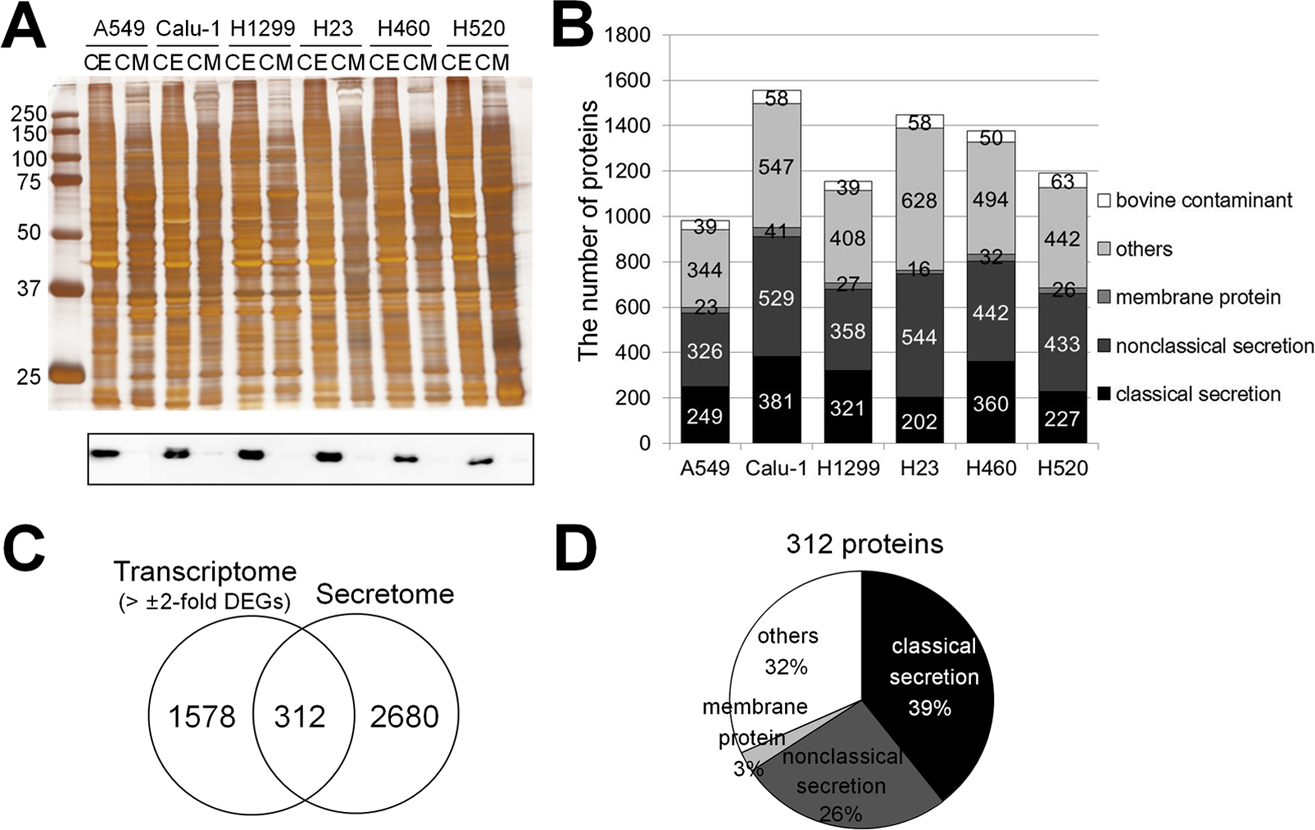
Analyses of conditioned media harvested from NSCLC cell lines. **(A)** Proteins (10 μg) in the conditioned media (CM) and cell extracts (CE) were analyzed by Western blot analysis using an anti-α-tubulin antibody. **(B)** The number of identified proteins in the cell secretome (FDR a 1%). Secretion pathways were predicted by SignalP, SecretomeP, and TMHMM. Bovine contaminants were distinguished using the human-FBS database. **(C)** Venn diagram of DEGs in tissues and identified proteins from cell secretomes. **(D)** Predicted secretion pathways of identified secretome proteins in all cell lines.

The secretomes of six lung cancer cell lines (A549, Calu-1, H1299, H23, H460, and H520) were profiled by LC-MS/MS (Fig 1). The main purpose of this analysis was to identify proteins that might be secreted from NSCLC cancer cells. As a negative control, α-tubulin was examined in cell secretomes by Western blot analysis. The cytoskeleton protein was clearly detected in cell extracts but not in conditioned media (Fig 3A), indicating that our secretome was rarely contaminated with intracellular proteins released from ruptured cells. In total, 2,992 non-redundant human proteins were identified from the six cell lines, among which 942, 1498, 1114, 1390, 1328, and 1128 proteins were from A549, Calu-1, H1299, H23, H460, and H520, respectively (Fig 3B and S2 Table).

### Integration of DEGs and cell secretome data

The DEGs identified from the public gene expression data were integrated with the proteins obtained by profiling cancer cell secretomes (Fig 1). After combining the 1,890 DEGs with the 2,992 secreted proteins, 312 proteins overlapped between the two lists (141 up-regulated DEGs and 171 down-regulated DEGs; Fig 3C). Since these 281 genes/proteins showed quantitative changes in cancer tissues compared to their levels in normal tissues and were also present in the cell secretome, we hypothesized that these proteins would be appropriate lung cancer biomarkers in plasma/serum. Therefore, these 312 proteins were considered as potential marker candidates for lung cancer.

The 312 proteins were further analyzed using bioinformatics programs designed to predict protein secretion pathways and 68% (213 of 312 human proteins) were expected to be secreted via various secretory pathways (Fig 3D and S2 Table). Briefly, based on the SignalP program, 39% of the proteins were secreted via classical secretory pathways based on the presence of a signal peptide (D-cut-off values for SignalP-noTM networks > 0.45 or SignalP-TM networks > 0.5 as the default cut-off for signal peptide = ‘Yes’) [28]. The SecretomeP 2.0 program predicted that 26% of the proteins were released via non-classical secretory pathways (SignalP signal peptide = ‘No; and SecretomeP score > 0.5) [29]. An additional 3% of the proteins were determined to be integral membrane proteins through TMHMM [30]. Of these, 259 proteins (83%) are reported as plasma proteins in PPD, among which 7.4% (23 proteins) were also evident in MRM-MS spectra.

### Detection of candidate proteins in plasma by LC-MS/MS analysis

**Fig 4:**
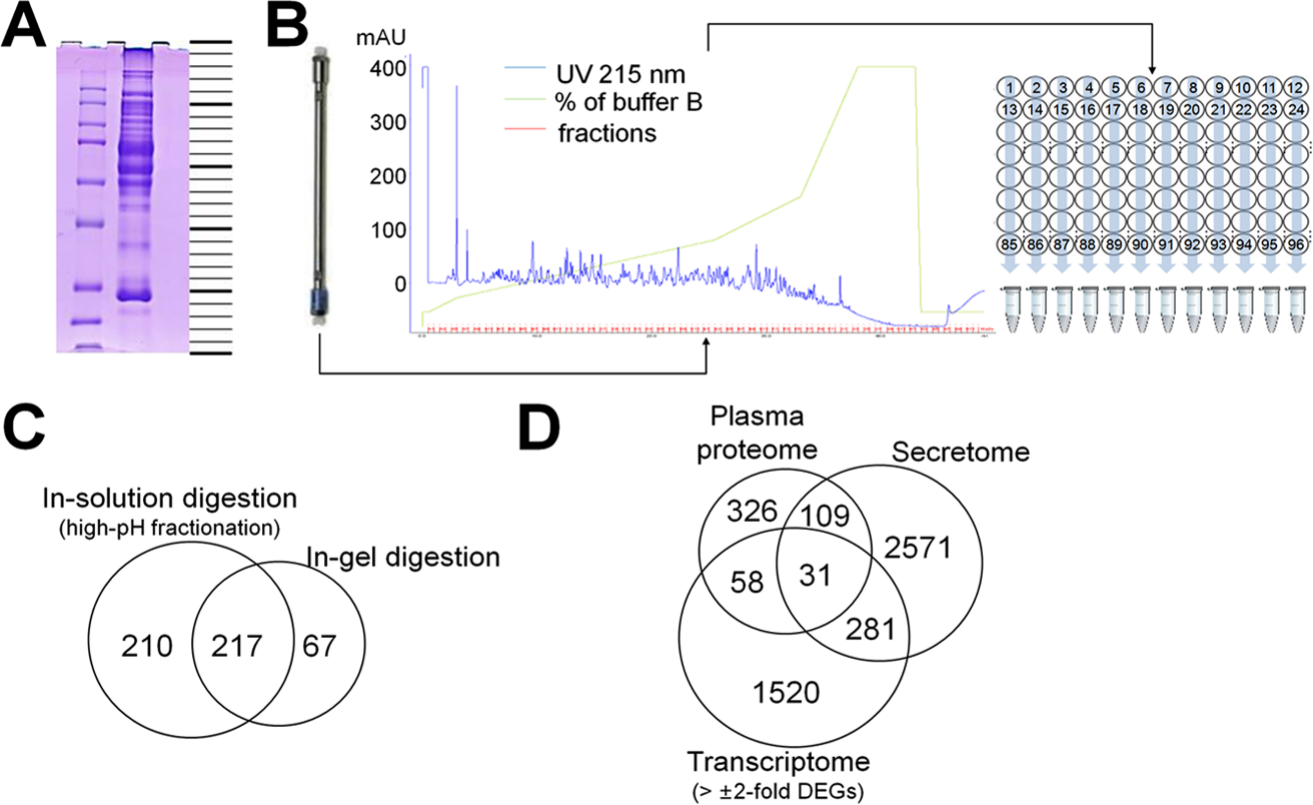
Analyses of proteomes from pooled plasma by mass spectrometry. **(A)** SDS-PAGE (protein 10 μg) of plasma pooled from 10 healthy control patients and 10 lung cancer patients, divided into 25 fractions. **(B)** Schematic diagram of the high-pH RPLC fractionation (protein 10 μg) setup. The eluates were combined by column (1–12 columns, 12 fractions). The surrogate peptides were monitored by measuring the UV absorbance of the eluates at 215 nm. **(C)** Venn diagram of the number of proteins identified by GeLC-MS/MS and high-pH RPLC fractionation. **(D)** Venn diagram of the number of analyzed molecules among DEGs, secretomes, and plasma proteome.

We next assessed the detectability of the 312 proteins in human plasma or sera (Fig 1). A sample prepared by combining the plasma from 10 lung cancer patients and 10 healthy persons was depleted of the top 12 abundant proteins and the resultant protein samples were analyzed by LC-MS/MS after fractionation via two independent methods, partly by GeLC (25 gel pieces shown in Fig 4A) and partly by in-solution trypsin digestion followed by high-pH fractionation (12 fractions shown in Fig 4B). All of the LC-MS/MS runs were performed with a predefined inclusion list. The inclusion list contained the m/z values of proteotypic peptides for some of the proteins that corresponded to DEGs and also detected in the secretome. In total, 524 non-redundant proteins were identified (Fig 4C and S3 Table) from this plasma proteome profiling, 314 were by GeLC, 457 by high-pH fractionation, and 247 by both. From the 312 candidate proteins, 31 proteins were finally selected based on integrative analyses combining publically available gene-expression data, cancer cell secretomes, and plasma proteomes of lung cancer patients (Fig 4D).

### Optimization of LC-MRM-MS for the detection of candidate proteins

The protein candidate list was narrowed down by applying several criteria at both protein and peptide levels. We first removed proteins with less than 10 PSMs. This is because, in general, the lower the PSM value, the less detectable in the MRM, and our proteome profiling conditions fitted to that cutoff according to previous experience. Some obviously unlikely proteins such as keratins and pregnancy zone protein were also removed. The proteotypic peptides and MRM transitions for the remaining proteins were chosen based on the results of plasma proteome profiling. Sequences of 6–20 amino acids in the Uniprot database (released as of 2014.02) and unique doubly charged tryptic peptides were preferentially selected. Peptides containing prolines at P2 (xxxxPK/R) or P3 (xxxxPxK/R) sites, proline after the tryptic site, basic amino acids at the P1’ site, and known single amino acid polymorphisms (SAPs) or post-translational modifications were excluded from the lists [13]. Finally, 39 peptides of the 8 proteins were selected as targets for LC-MRM-MS.

Tandem mass spectra of the 39 chosen peptides were retrieved from the Global Proteome Machine (GPM) and National Institute of Standards and Technology (NIST) databases and used to compose MRM transitions. Since we intended to exclude the affinity-based depletion process from the MRM analysis to maintain high reproducibility, we had to evaluate the feasibility of accessing the peptides via MRM in whole plasma/serum without prior immunodepletion. We monitored 191 transitions of 39 peptides by employing 3- to 14-min scheduled LC-MRM runs [33]. Eight peptides of five proteins were clearly detected and thus considered to be detectable and quantifiable marker candidates to include in the following validation process by LC-MRM-MS. Accordingly, we synthesized heavy isotope-labeled standards for the 8 target peptides (S4 Table), and optimized instrumental parameters such as declustering potential (DP), collisional energy (CE) and collision cell exit potential (CXP) [33].

### Baseline and demographic characteristics of serum samples

Baseline and demographic characteristics are listed in Table 2. For measurement of biomarkers by MRM-MS, 46 individuals were enrolled in this study. The 23 subjects enrolled in the control group visited our hospital for regular health screening tests. No subject in the control group had any previous history of malignant disease. The mean age of the control group was 56.5 ± 14.9 years. Fourteen subjects were male (56%) and six had a smoking history (26%). Five persons had HTN (22%) and one person had DM (4%). The 23 persons, enrolled in the NSCLC group, included 16 (70%) adenocarcinoma patients and 7 (30%) squamous cell carcinoma patients. The mean age of the NSCLC group was 60.3 ± 9.8 years; 10 patients (43%) were male and 6 patients (26%) had a smoking history. Eight persons had HTN (35%) and four had DM (21%).

**Table 2:** Demographics of control and non-small cell lung cancer (NSCLC) groups.

	MRM-MS			ELISA		
	Control	NSCLC^a^	p-value	Control	NSCLC^a^	p-value
Number	23	23		50	50	
Age	56.5±14.9	60.3±9.8	0.66^d^	56.9±10.2	59.2±8.4	0.23^d^
Gender (M/F)	14/9	10/13	0.24^e^	26/24	17/33	0.07^e^
Smoke (Yes/No)	6/17	6/17	1.00^e^	13/37	14/36	0.82^e^
HTN^b^ (Yes/No)	5/18	8/15	0.33^e^	12/38	19/31	0.13^e^
DM^c^ (Yes/No)	1/22	4/19	0.35^e^	7/43	8/42	0.78^e^

^a^ NSCLC–the MRM group includes 16 ADCs and 7 SQCs and the ELISA group 40 ADCs and 10 SQCs. (ADC: adenocarcinoma, SQC: squamous cell carcinoma)

^b^ HTN: hypertension;

^c^ DM: diabetes mellitus

^d^ Mann-Whitney test (NSCLC *vs*. control)

^e^ Pearson Chi-Square test (NSCLC *vs*. control)

The sample size computed from the mean and SD (Table 3) of the two groups in the BCHE and GPx3 MRM data (α = 0.05, 1-β = 0.80) were 56 and 78, respectively. Therefore, an independent set of control (n = 50) and NSCLC (n = 50) samples (i.e., 100 in total) was selected for the ELISA measurement of two biomarkers. The mean age of the control group was 56.9 ± 10.2 years. Twenty-six were male (52%), and 13 had a smoking history (26%). Twelve persons had HTN (24%) and seven had DM (14%). The NSCLC group (n = 50) included 40 (80%) adenocarcinoma patients and 10 (20%) squamous cell carcinoma patients. The mean age of the NSCLC group was 59.2 ± 8.4 years; 17 patients (34%) were male and 14 (28%) had a smoking history. Nineteen persons had HTN (38%) and eight had DM (16%).

**Table 3:** Values of four biomarker candidates in the control and NSCLC^a^ groups by MRM-MS and ELISA.

	MRM-MS			ELISA		
(μg/mL)	Control	NSCLC^a^	p-value^b^	Control	NSCLC^a^	p-value^b^
BCHE	4.87 ± 1.27	3.91 ± 1.17	<0.05	4.50 ± 0.93	4.04 ± 1.12	<0.05
C7	40.72 ± 12.79	32.61 ± 14.00	0.11			
CP	2006.94 ± 538.75	1934.31 ± 631.82	0.49			
GPx3	11.20 ± 2.27	9.79± 1.98	<0.05	13.79 ± 5.25	9.01 ± 4.27	<0.01

^a^ NSCLC: non-small cell lung cancer

^b^ Mann-Whitney test (NSCLC *vs*. control)

### Validation of marker candidates for NSCLC by MRM-MS assay

**Fig 5:**
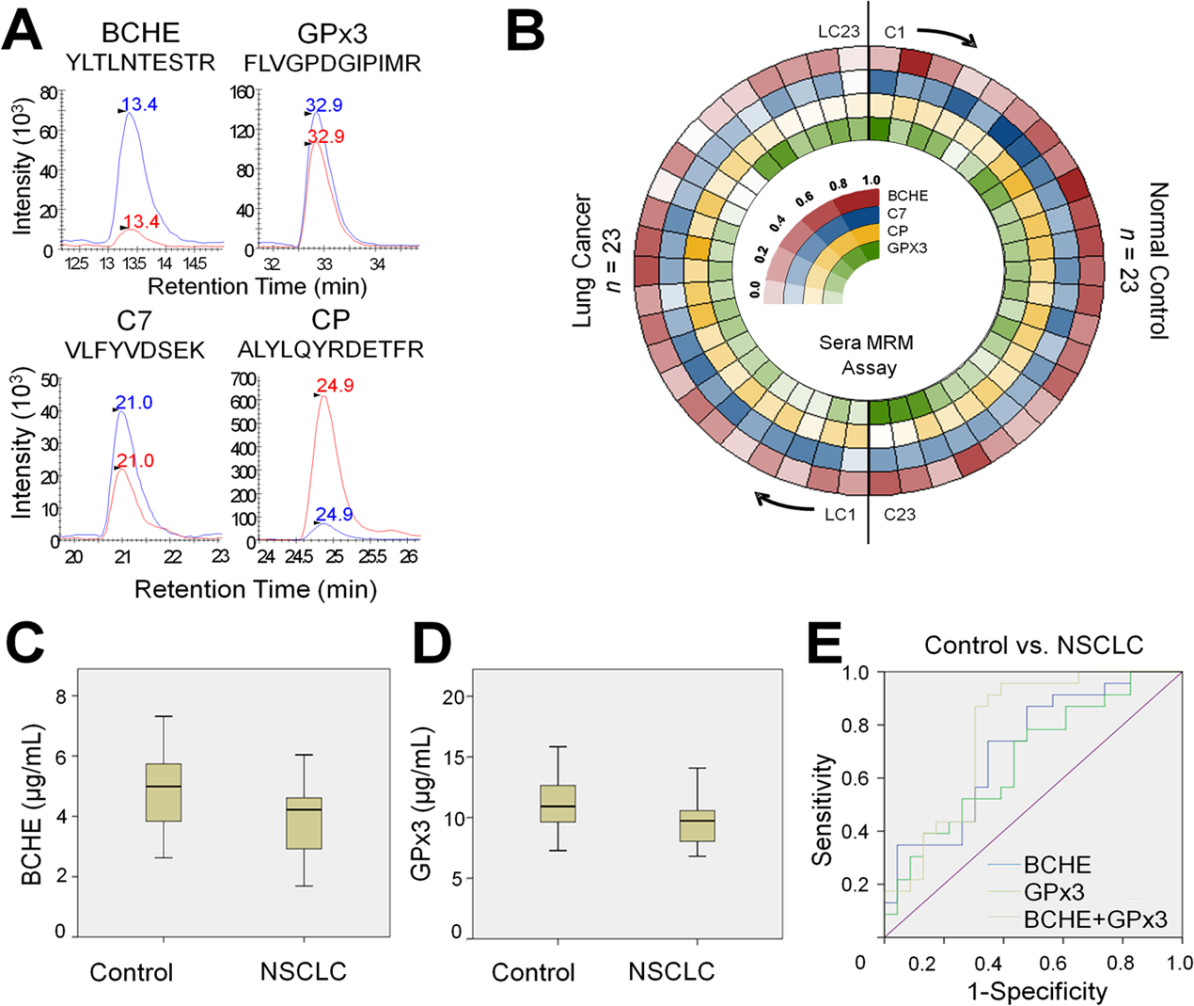
Systematic evaluation of serum MRM assays. **(A)** Total ion chromatogram (TIC) of endogenous (red) peptides and their respective SIS peptides (blue). **(B)** Circular heatmap of the relative expression of four proteins in the two groups. The 46 clinical samples are shown in the circular heat map, clockwise from the top: 23 controls and 23 NSCLCs (16 adenocarcinomas and 7 squamous cell carcinomas). Indexing was followed by the sequence of LC-MRM runs. **(C)** Serum levels of BCHE in the control and NSCLC groups **(D)** Serum levels of GPx3 in the control and NSCLC groups. **(E)** ROC curves of BCHE, GPx3, and the combination of the two proteins. NSCLC: non-small cell lung cancer

Through MRM-MS assays, we monitored eight surrogate peptides of five target proteins in the abovementioned samples without immunodepletion or fractionation. All samples were randomized and then analyzed sequentially. In addition, seven randomly chosen samples (five from healthy controls, and two from adenocarcinoma patients) were analyzed in triplicate to evaluate the reproducibility and precision of our analytical procedure (S1A Fig). The XICs of the endogenous peptides were compared to those of the corresponding SIS peptides to obtain final quantitation values (Fig 5A). Of the eight peptides that were monitored by MRM, one peptide, GGH.YYIAASYVK, showed very low signal-to-noise ratio of less than three in more than 50% of both the normal and NSCLC sera samples and the CV of two triplicate runs exceeded 30%. The CVs of the remaining seven peptides were below 20% with a median of 3.8%. For three proteins, BCHE, CP, and GPx3, for which two peptides were monitored simultaneously, the peptide showing the higher MRM signal was used in the final protein-level quantitation. The only remaining protein, C7 had only one representative peptide (S4 Table). The relative standard deviations (RSDs) (%) of the retention times of the four quantotypic peptides were less than 6% (S1B Fig). The levels of the four target proteins are displayed in Fig 5B as a circular heat map in the order of sample assessment. After obtaining these results, we performed further statistical analysis (S5 Table).

The levels of the four biomarker candidates were compared between the two groups (control and NSCLC) using the Mann-Whitney test (Table 3). The only two proteins (BCHE and GPx3) whose levels were significantly different between the control and NSCLC groups were selected. The mean BCHE levels of the control and NSCLC groups were 4.87 ± 1.27 μg/mL and 3.91 ± 1.17 μg/mL, respectively (p < 0.05); the GPx3 levels of the control and NSCLC groups were 11.20 ± 2.26 μg/mL and 9.79 ± 1.98 μg/mL, respectively (p < 0.05) (Fig 5C and 5D and Table 3). With the selected proteins BCHE and GPx3, multivariate logistic regression analysis was done to further test the association of biomarkers with the risk of lung cancer (S6 Table). This test showed that both biomarkers statistically significantly decrease the risk of lung cancer: for BCHE, Exp(β) = 0.525 (p < 0.05) and for GPx3 Exp(β) = 0.735 (p = 0.059). Based on the ROC analysis, the AUC for BCHE was 0.713 (95% confidence interval (CI): 0.563–0.862, p < 0.05). The AUC of GPx3 was 0.673 (95% CI: 0.517–0.828, p < 0.05). When these two values were combined, the AUC increased to 0.773 (95% CI: 0.631–0.915, p < 0.01) (Fig 5E).

### Validation test with ELISA-based quantification of GPx3 and BCHE

**Fig 6:**
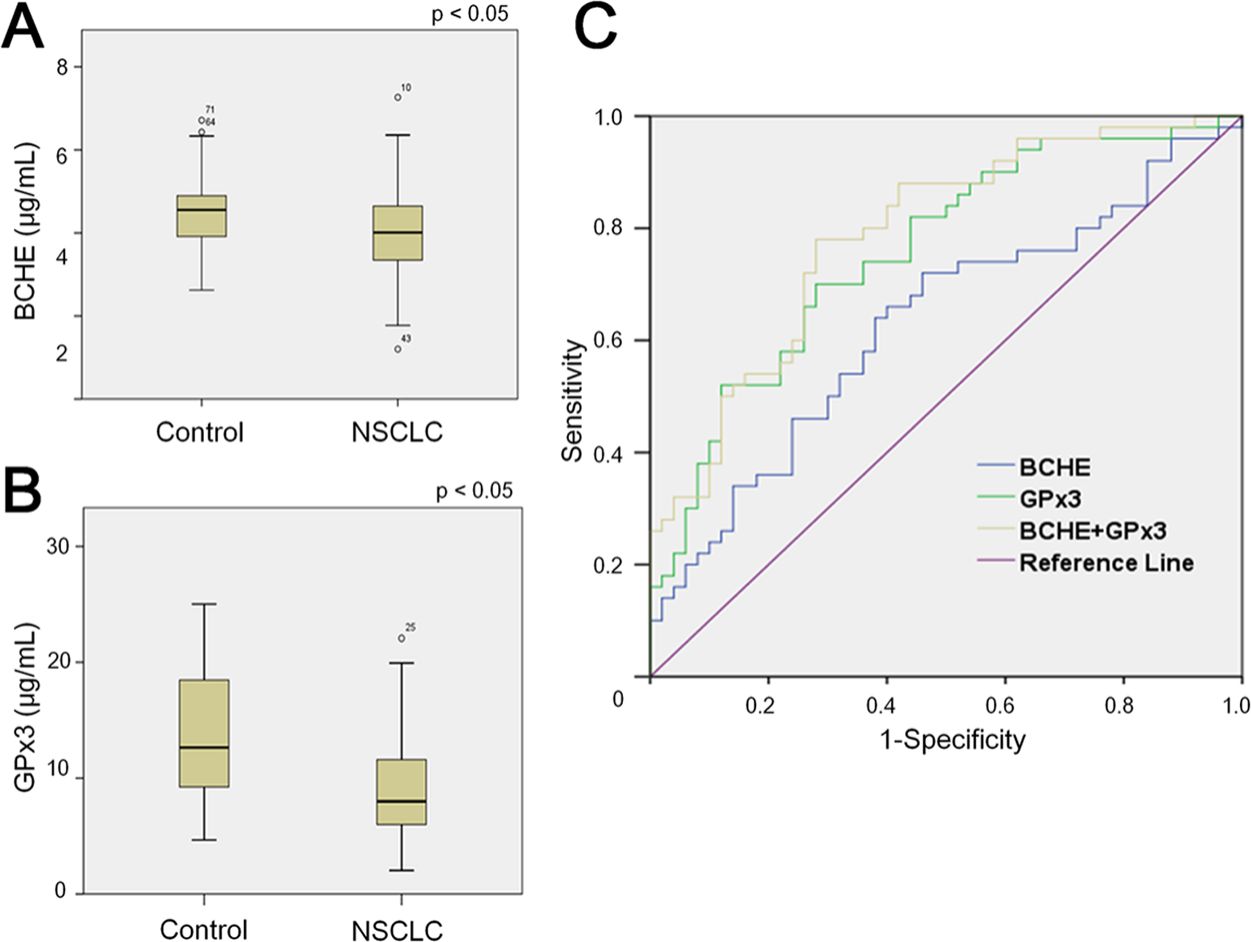
Quantitation of the two proteins in an independent set of the control (n = 50) and NSCLC (n = 50) samples using ELISA. Serum levels of **(A)** BCHE and **(B)** GPx3 in the control and NSCLC groups. **(C)** ROC curves of GPx3, BCHE, and a combination of the two proteins. NSCLC: non-small cell lung cancer

The two biomarkers selected by MRM (BCHE and GPx3) were validated by ELISA with an independent group of control and patient (NSCLC) samples (S6 Table). The levels of BCHE and GPx3 were compared between the control and NSCLC groups by the Mann-Whitney test (Table 3). The BCHE level of the control group was significantly different from that of the NSCLC group (p < 0.05). The mean BCHE levels of the control and NSCLC groups were 4.50 ± 0.93 μg/mL and 4.04 ± 1.10 μg/mL, respectively (Fig 6A and Table 3). The GPx3 level of the control group was also significantly different from that of the NSCLC group (p < 0.01); the mean GPx3 levels of the control and NSCLC groups were 13.79 ± 5.25 μg/mL and 9.01 ± 4.27 μg/mL, respectively (Fig 6B and Table 3). Multivariate logistic regression analysis was done to further test the association of BCHE and GPX3 with the risk of lung cancer (S7 Table). The result was that they do statistically significantly decrease the risk of lung cancer: for BCHE, Exp(β) = 0.571 (p < 0.05) and for GPx3 Exp(β) = 0.803 (p < 0.01). ROC curves were constructed for GPx3 and BCHE in the validation population. The AUC for BCHE was 0.630 (95% CI: 0.520–0.740, p < 0.05); the AUC for GPx3 was 0.759 (95% CI: 0.665–0.852, p < 0.01). In the compound model of GPx3 and BCHE, the AUC was 0.788 (95% CI: 0.700–0.876, p < 0.01) (Fig 6C).

## Discussion

Quantitative analyses of gene expression combined with profiling of cell secretomes might improve discovery of disease-related markers. In this study, we started with the gene expression data of nine NSCLC transcriptome datasets from the GEO database [14–22]. Quantitative information on the differential gene expressions of tumor tissues and normal tissues were then merged with cancer cell secretome data and patient’s plasma proteome data. We hypothesized that if any protein found in the lung cancer cell secretome showed quantitative differences in its gene expression between normal and NSCLC tissues, it might be a suitable serological marker. Although protein abundance is not a simple function of DNA copies and mRNA levels, genes that are dysregulated at the level of DNA or RNA are also generally dysregulated at their protein level [3]. This supports the validity of our strategy used to identify cancer biomarkers. A limitation of our study is that we used the cell line secretome to infer the cancer secretome in circulation. It is difficult to assess the degree of relevance between the *in vitro* secretome and the *in vivo* cancer secretome. However, several sampling methods such as tissue explant, 3-dimensional cell culture, and coculture have been developed, which more closely reflect *in vivo* conditions [34]. These methods will thus enable evaluation of the possible crosstalk between cancer cells and their surroundings.

Although MRM-MS and ELISA were not performed using the same sample set, the measured concentration scales in serum and the down-regulated patterns in NSCLC patients compared to healthy controls were similar, regardless of differences between experimental techniques. Most validation processes used in the discovery of marker candidates and diagnostic applications in clinical practice have employed antibody-based ELISA methods. However, such methods suffer from throughput limitations, as each assay can monitor only one protein at a time and requires well-qualified antibodies. The emerging development of MRM analysis has gradually fulfilled the steadily rising demand for quantitative proteomic data, due to its high-throughput capability and low cost compared to immunoassay-based approaches [33, 35–38]. Recently, some groups have published MRM-based marker validation in the plasma/serum of lung cancer patients [39, 40]. Li et al. presented a 13-protein, blood-based classifier that differentiates malignant nodules from benign lung nodules as a diagnostic tool that would obviate invasive biopsies on benign lung nodules [40]. In our study, 46 serum samples representing two sample groups (23 NSCLC patients and 23 controls) were analyzed by mass spectrometry, in which eight surrogate peptides from five proteins were quantified by MRM analysis. Peptides were selected based on their observation in standard LC-MS analyses of plasma. We then applied stringent peptide selection criteria, such as the length of the peptide sequence, charge state and position of specific amino acids, and the number of missed cleavage sites to identify the peptides most suitable for use as biomarkers. Optimized sample preparation and an analysis workflow were developed and successfully applied to the relative quantification of candidate biomarkers in the serum of patients. In addition, without fractionation of the human serum digests, surrogate peptides of marker candidate proteins were detectable via MRM–MS. Finally, one peptide per protein was selected according to its spectral intensity to minimize any statistical bias.

Glutathione peroxidase, anti-oxidant scavenging ROS (reactive oxygen species), is known to play an important role in signaling of molecules in cell physiology [41]. The selenocysteine-containing protein GPx3 accounts for the only extracellular form of glutathione peroxidase activity in blood [42]. GPx3 is mainly synthesized in the proximal tubules of the kidney and is usually down-regulated in cancer tissues [43]. It has been reported that its activity is significantly reduced in the blood of patients with breast, gastric, prostate, thyroid, and colorectal cancer [44]. It has also been reported that GPx3 promotes hypermethylation, and its down-regulation is commonly observed in human cancers [45–47]. In a previous study, we found that the mean serum GPX3 levels were lower in the lung cancer group compared to control group [48]. And other recent studies showed the involvement of GPx3 in the pathogenesis of other human cancers such as gastric, ovarian and prostate cancer [45, 49, 50]. Since GPx3 is highly expressed in healthy tissues, this suggests it may exhibit tumor suppressive activity.

Vertebrates contain two cholinesterases, acetylcholinesterase (AChE) and butyrylcholinesterase (BChE). AChE prefers acetylcholine as a substrate and its function is critical for terminating cholinergic responses [51]. Experimental evidence supports the involvement of cholinesterase in cancers such as leukemia, ovarian cancer, brain tumors, breast cancers, and lung cancers. Martinez-Moreno reported that BCHE activity decreased by 40–50% in adenocarcinoma, squamous cell carcinoma, and large cell carcinoma extracts [52]. However, in the reports of Zanini et al., BCHE activity was not altered in lung cancer patients when compared to a control group [51]. Kaniaris et al. also examined cholinesterase levels in cancer patients. They found that serum cholinesterase activity was significantly lower in patients with cancer in correlation with the extent of disease [53].

To insist on our strategy for biomarker discovery to be right, it may be necessary to confirm the two genes selected on their mRNA expression level in cancer/normal tissue are expressed in protein level concordantly. We looked, to address this issue, into the expression patterns of the two proteins in the Human Protein Atlas (http://www.proteinatlas.org/search/GPx3 and http://www.proteinatlas.org/search/BCHE). According to the data on this web site, the expression levels of GPx3 in lung cancer and adjacent normal tissue are Not-detected (level 1) and Low (level 2), respectively. And the BCHE expression level is Medium (level 3) to Low (level 2) in lung cancer and Low (level 2) to Not-detected (level 1) in adjacent normal tissue. For the case of BCHE, it could look like the BCHE protein was locally more expressed in cancer tissue. However, please see other reference [52], supporting that the BCHE activity (enzyme activity) was lower in ADC (5.85 ± 3.20 mU/mg) and SCC (4.49 ± 2.30 mU/mg) when compared to the adjacent non-cancerous tissue (9.56 ± 3.38 mU/mg in ADC; 6.56 ± 4.09 mu/ml in SCC). This result matched with the fact that the two genes, first selected on the basis of their expression in mRNA level between cancer and its adjacent normal tissue, are displaying their protein expression concordantly in tissue and thereafter in blood.

Our results from MRM-MS and ELISA clearly show that BCHE and GPx3 levels were significantly lower in the NSCLC group than in the control group. In addition, we investigated whether there is any correlation between lung cancer stage and biomarker levels in the MRM and ELISA data (S8 Table). The test showed that BCHE and GPx3 have no statistically significant correlation with the stages of NSCLC patients. Next, we further looked into any association of smoking history and biomarker levels in NSCLC. According to previous publications, the tissue activity of BCHE was higher in ADC than in SQC patients [52] and the blood level of GPx3 was lower in ADC than in SQC patients [48]. Considering together the fact that the number of cell type (ADC and SQC) were not matched in the groups of smoke and non-smoke, we therefore performed cell type-stratified analysis of biomarker levels in NSCLC (S9 Table). The result was that there was no significant difference in the level of BCHE and GPx3 in the groups of smoke and non-smoke. We nevertheless think that it is necessary to confirm this result because the numbers of samples enrolled in this test.

With the advent of screening tests that include chest computerized tomography (CT), the chances of discovering benign lung nodules are increasing. In particular, sub-centimeter lung nodules are difficult for differential diagnoses of cancer. There are some guidelines for the management of lung nodules that are detected incidentally. However, most of these guidelines are based on findings associated with chest CT images. In the presence of suitable biomarkers for NSCLC, more accurate diagnoses of cancer are possible, leading to improved therapeutic results [54–57]. This pipeline finally led to the validation of two marker candidates for NSCLC. As per our experimental observations, if persons with benign lung nodules can be included in the control group, the discriminating power of these biomarkers will be more meaningful clinically, and such analysis can also be used as a supplementary tool with lung cancer screening tests via CT analysis. The validation process described herein was however conducted with a retrospective selection of limited-sized samples (S2 Fig). A prospective study including lung nodules is still required to validate further the utility of biomarkers for clinical use. Moreover, since the effect size (computed from mean and SD) between the control and NSCLC groups in the BCHE and GPx3 data were not high, the sensitivity and specificity (i.e., AUC in ROC analysis) of the two biomarkers are not so impressive. This indicates that even though they are statistically significant classifier between Control and NSCLC, BCHE and GPx3 may not be too robust to be applicable right on the clinical field soon. We think that it is needed to conduct a more comprehensive study with an independent patient cohort with appropriate sample size.

## Supporting information

S1 Fig Sequence and CVs for retention time of LC-MRM-MS analysis. (TIF)

S2 Fig Dot (scatter) presentation of MRM and ELISA data. (TIF)

S1 Table Data of DEGs of lung cancer tissues obtained by GEO data analysis. (XLSX)

S2 Table Data of identified NSCLC cell secretome proteins. (XLSX)

S3 Table Data of profiled proteins in pooled plasma. (XLSX)

S4 Table List of eight target peptides for LC-MRM-MS. (XLSX)

S5 Table Data of four target proteins in MRM-MS assays. (XLSX)

S6 Table Data of BCHE and GPx3 proteins in ELISA assays. (XLSX)

S7 Table Multivariate logistic regression analysis of BCHE and GPx3 for NSCLC. (DOCX)

S8 Table Correlation analysis between BCHE / GPx3 and lung cancer stage. (DOCX)

S9 Table Cell-type stratified analysis of BCHE and GPx3 between smoke vs. non-smoke. (DOCX)
