# Supplementary figures and images for: Integrative analysis for the discovery of lung cancer serological markers and validation by MRM-MS
Source: PLoS One. 2017 Aug 24;12(8):e0183896. doi: 10.1371/journal.pone.0183896 (PMC5570484; doi:10.1371/journal.pone.0183896)

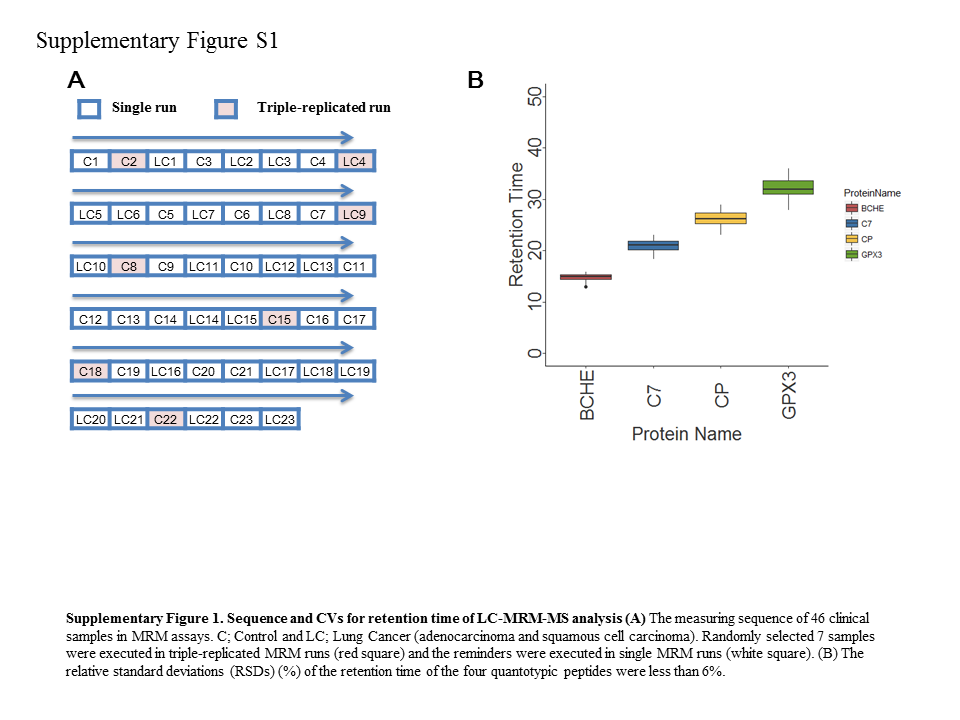

Supplement: S1 Fig — (TIF) [file pone.0183896.s001.tif]

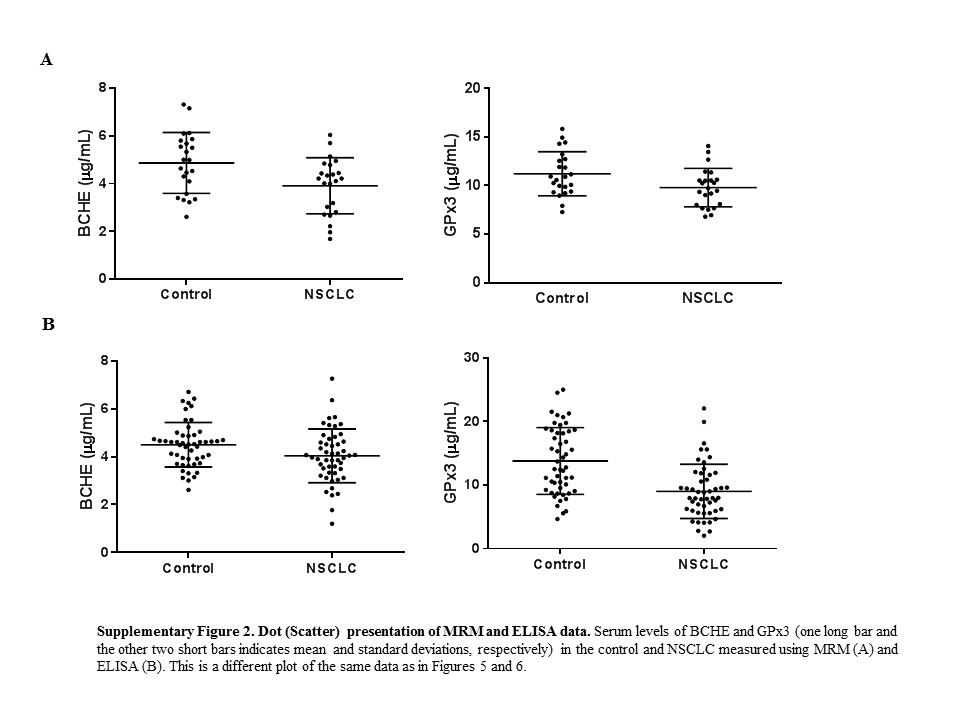

Supplement: S2 Fig — (TIF) [file pone.0183896.s002.tif]
