# Supplementary material for: Integrative analysis for the discovery of lung cancer serological markers and validation by MRM-MS
Source: PLoS One. 2017 Aug 24;12(8):e0183896. doi: 10.1371/journal.pone.0183896 (PMC5570484; doi:10.1371/journal.pone.0183896)
Supplement: S7 Table — (DOCX) [file pone.0183896.s009.docx]

**Supplemental Table S7. Multivariate logistic regression analysis of BCHE and GPx3 for the risk of lung cancer in the MRM(A) and ELISA(B) data. β is the estimated regression coefficient.**

(A) MRM

| Factor | Exp(β) | *p* |
| --- | --- | --- |
| BCHE | 0.525 | 0.027 |
| GPx3 | 0.735 | 0.059 |

(B) ELISA

| Factor | Exp(β) | *p* |
| --- | --- | --- |
| BCHE | 0.571 | 0.020 |
| GPx3 | 0.803 | <0.001 |
