# Supplementary material for: Integrative analysis for the discovery of lung cancer serological markers and validation by MRM-MS
Source: PLoS One. 2017 Aug 24;12(8):e0183896. doi: 10.1371/journal.pone.0183896 (PMC5570484; doi:10.1371/journal.pone.0183896)
Supplement: S8 Table — (DOCX) [file pone.0183896.s010.docx]

**Supplemental Table S8. Spearman correlation analysis of BCHE and GPx3 for the association of lung cancer stage^a^ in the MRM (A) and ELISA(B) data.**

| Factor | Correlation coefficient^b^ | *p* |
| --- | --- | --- |
| MRM |  |  |
| BCHE | -0.066 | 0.765 |
| GPx3 | 0.381 | 0.073 |
| ELISA |  |  |
| BCHE | -0.134 | 0.352 |
| GPx3 | 0.248 | 0.083 |

^a^ The number of patients for MRM analysis in stages I, II, III and IV are 14, 2, 3 and 4, respectively; the number for ELISA analysis in stages I to IV are 14, 0, 13 and 23, respectively.

^b^Correlation coefficient – Spearman’s rho.
