# Supplementary material for: Integrative analysis for the discovery of lung cancer serological markers and validation by MRM-MS
Source: PLoS One. 2017 Aug 24;12(8):e0183896. doi: 10.1371/journal.pone.0183896 (PMC5570484; doi:10.1371/journal.pone.0183896)
Supplement: S9 Table — (DOCX) [file pone.0183896.s011.docx]

**Supplemental Table S9. Cell type–stratified analysis of BCHE and GPx3 levels between smokers and non-smokers in NSCLC.**

(A) MRM

|  |  | ADC |  |  | SQC |  |
| --- | --- | --- | --- | --- | --- | --- |
| μg/mL | smoke (n=3) | no-smoke (n=13) | p-value | Smoke (n=3) | No-smoke (n=4) | p-value |
| BCHE | 3.98±1.18 | 4.13±0.90 | 0.80 | 3.27±1.05 | 2.89±1.88 | 0.34 |
| GPx3 | 9.33±1.27 | 10.16±2.29 | 0.56 | 10.01±40.73 | 8.73±2.00 | 0.35 |

(B) ELISA

|  |  | ADC |  |  | SQC |  |
| --- | --- | --- | --- | --- | --- | --- |
| μg/mL | smoke (n=9) | no-smoke (n=31) | p-value | Smoke (n=5) | No-smoke (n=5) | p-value |
| BCHE | 3.70±0.71 | 4.44±1.00 | 0.05 | 2.50±1.19 | 3.59±0.75 | 0.12 |
| GPx3 | 8.70±2.92 | 7.85±3.89 | 0.55 | 14.47±5.62 | 11.25±2.69 | 0.28 |
